# Breast cancer in 30-year-old or younger patients: clinicopathologic characteristics and prognosis

**DOI:** 10.1186/s12957-015-0462-4

**Published:** 2015-02-12

**Authors:** Yongqiang Yao, Mingqian Cao, Hong Fang, JiPing Xie

**Affiliations:** Affiliated Zhongshan Hospital of Dalian University, Dalian, 116001 China

**Keywords:** Age, Breast cancer, Prognosis, Survival

## Abstract

**Background:**

The number of 30-year-old or younger patients with breast cancer is increasing. The aim was to describe the clinicopathological features and prognosis of 30-year-old or younger patients with breast cancer.

**Methods:**

We reviewed the records of 1,406 consecutive patients aged ≤50 years with first diagnosis of invasive breast cancer referred to surgery from March 2001 to March 2009. A total of 105 patients were aged ≤30 years (group I) and 1,301 were aged 31–50 years (group II).

**Results:**

Compared with patients of group II, patients of group I had a higher percentage of tumors classified as estrogen receptors (ER) negative (*P* < 0.001) and progesterone receptors (PR) negative (*P* = 0.043), with a Ki-67 labeling index ≥20% of the cells (*P* = 0.011). There was no difference between the two groups for pT and pN, histology, endocrine therapy, and chemotherapy. The 5-year survival of group I was 67.5% as compared with 75.3% for group II (*P* = 0.003).

**Conclusions:**

Compared with patients aged between 31 and 50 years, patients aged ≤30 years have a greater chance of having an endocrine-unresponsive tumor and a significantly poor prognosis.

## Background

In China, breast cancer is the most commonly diagnosed cancer in women. Breast cancer rarely occurs in young women. Breast cancer before the age of 50 is relatively uncommon, nevertheless, 15%–25% of all women diagnosed with breast cancer are in their thirties or forties [1]. Furthermore, about 2%–4.8% of the patients with breast cancer are <35 years old at diagnosis [1,2]. In Asian, about 9.5%–12% of the patients with breast cancer are <35 years old at diagnosis [3]. Breast cancer at a very young age has been reported to have a more aggressive biological behavior compared with the disease in older patients. Specifically, in previously published reports, the tumors progress faster, present with higher grade, and are more often estrogen receptors (ER) negative than the tumors in older patients [1,4,5].

One population-based study revealed a relationship between age at diagnosis and risk of death, with the youngest having a higher risk than patients of intermediate age [6]. Furthermore, a review of the National Cancer DataBase has shown that younger patients had more advanced disease at diagnosis and a poorer 5-year survival than older premenopausal patients [7]. However, other studies have revealed that age is not related to overall or disease-free survival after adjustment for other prognostic variables [8,9]. Several authors have even reported that young patients have better survival [10,11]. In addition, data on treatment effects are largely dependent upon older series collected over several years and extrapolation of data from older age cohorts. Staging procedure and immunohistochemical determination of ER and progesterone receptors (PR) are features that underwent a more or less substantial change in recent years.

In the present study, we have aimed to investigate the most recently available details of clinicopathological features and prognosis of very young patients (≤30 years of age) with breast cancer.

## Methods

### Patients

We analyzed data from 3,152 patients with histologically proven breast cancer operated on in our institution between March 2001 and March 2009. Information on the patient’s pT stage, pN stage, ER level, PR level, Ki-67 level, histology, endocrine therapy, and chemotherapy were obtained from the hospital records. We divided 3,152 patients into three age groups ≤30 years, 31–50 years, and >50 years. In the present study, we compared clinicopathological features and prognosis in two groups of patients: age ≤30 years and age between 31 and 50 years. Informed consent had been obtained, and the Ethics Committee of Dalian University approved this study.

Patient follow-up lasted until death or the cutoff date of March 31, 2014. Generally, patients return every 3 months for the first year, every 6 months for the next 2 years, and after 3 years every 12 months for life. For all patients, at the time of the last follow-up, 122 patients (3.9%) had been lost to follow-up. Only patients who died of breast cancer were regarded as tumor-related death cases.

### Statistical analysis

Statistical comparisons for significance were performed with the chi-square test for discrete variables. The Kaplan-Meier method was used for calculating cumulative survival rate, and the difference between groups was assessed by using the log-rank test. The accepted level of significance was *P* < 0.05. All data analysis was performed using the SPSS for Windows, Version 13.0 software package.

## Results

We analyzed data from 3,152 patients divided into three age groups: ≤30 years (group I: *n* = 105), 31–50 years (group II: *n* = 1301), and >50 years (groups III: *n* = 1746). We compared clinicopathological features and prognosis in two groups of patients: age ≤30 years and age between 31 and 50 years.

Table 1 shows the clinicopathological factors for group I as opposed to group II. There were statistical differences in ER level, PR level, and Ki-67 level between the two age groups. In group I, when compared with group II, there were higher percentages of tumors classified as ER negative (39.0% versus 21.1%, *P* < 0.001), PR negative (46.7% versus 36.7%, *P* = 0.043), with a Ki-67 labeling index ≥20% of the cells (71.4% versus 58.8%, *P* = 0.011) (Table 1).

**Table 1:** **Clinicopathological factors according to age group**

**Variables**	**Group I (age ≤30 years) (** ***n*** **= 105; %)**	**Group II (age between 31 and 50 years) (** ***n*** **= 1,301; %)**	***P*** **value**
pT stage			
pT1	55 (52.4)	754 (58.0)	
pT2	44 (41.9)	456 (35.0)	
pT3/4	6 (5.7)	91 (7.0)	0.361
pN stage			
pN0	43 (41.0)	561 (43.1)	
pN1	31 (29.5)	392 (30.1)	
pN2	17 (16.2)	172 (13.2)	
pN3	14 (13.3)	176 (13.5)	0.859
ER level			
Percentage ER <10%	41 (39.0)	275 (21.1)	
Percentage ER ≥10%	64 (61.0)	1,026 (78.9)	<0.001
PR level			
Percentage PR <10%	49 (46.7)	478 (36.7)	
Percentage PR ≥10%	56 (53.3)	823 (63.3)	0.043
Ki-67 level			
Percentage Ki-67 < 20%	30 (28.6)	536 (41.2)	
Percentage Ki-67 ≥ 20%	75 (71.4)	765 (58.8)	0.011
Histology			
Ductal	93 (88.6)	1,098 (84.4)	
Lobular	3 (2.9)	88 (6.8)	
Other	9 (8.6)	115 (8.8)	0.287
Endocrine therapy			
Yes	54 (51.4)	769 (59.1)	
No	51 (48.6)	532 (40.9)	0.124
Chemotherapy			
Yes	73 (69.5)	865 (66.5)	
No	32 (30.5)	436 (33.5)	0.525

No statistically significant difference was observed for the stage of disease at diagnosis [according to the tumor-node-metastasis (TNM)] [12], for pT and pN stage. In fact, pT1 was registered in 52.4% and 58.0% of the patients in the two groups, respectively. Similarly, 29.5% and 30.1% had one to three lymph nodes involved and 13.3% and 13.5% had ≥10 nodes in the two groups, respectively. For the histology, endocrine therapy, and chemotherapy, there was no significant difference between the two age groups.

The 5-year survival rates of patients, according to age, are shown in Table 2. The 5-year survival of group I was 67.5% as compared with 75.3% for group II (*P* = 0.003).

**Table 2:** **Five-year survival rates according to age group**

**Age (years)**	**Number of patients**	**5-year survival rate (%)**	***P*** **value**
≤30	105	67.5	
31–50	1,301	75.3	0.003

## Discussion

Breast cancer that develops at a young age is commonly considered to be different from that arising in older premenopausal patients. Tumors occurring in very young patients are reported to have a particularly aggressive biological behavior leading to a somewhat unfavorable prognosis, which was described extensively in the pre-adjuvant systemic therapy era [6]. Data of our study were derived from our hospital records with information on the most important prognostic variables such as tumor size, lymph node status, and histology. We could not evaluate the risk of recurrence according to age because our hospital records did not routinely collect this information. In the study, there was no difference between the two age groups for pT and pN, histology, endocrine therapy, and chemotherapy. Compared with patients aged between 31 and 50 years, patients aged ≤30 years have a greater chance of having an endocrine-unresponsive tumor and a significantly poor prognosis.

Because of the lower probability of having breast cancer at young age, both women and physicians might perform self-examination or clinical examination less often and minimize putative abnormalities [13]. Owing to the fact that the breast tissue of younger women is denser, it is more difficult to examine clinically and by mammography. Diagnosis is often delayed in young women, and cancers are larger and more advanced [4,8]. A more advanced stage at diagnosis may also reflect a rapidly growing tumor. Special endocrine and immunological factors and/or genetic differences more frequent in younger women who develop breast cancer may be linked to tumor invasiveness [14]. For breast cancer, the TNM stage proved to be the most significant independent prognostic factor for determining survival. In our study, there was no difference between the two age groups for pT and pN, thus not supporting previous data indicating more advanced disease in younger patients at diagnosis of operable disease. The result is similar to that of Colleoni et al. [1].

Several studies have shown that survival among young women is worse than that of older women [4,15,16]. However, in some of these studies, the observations are drawn from a single hospital and selection may have taken place during the process of referring patients to the hospital [15,16]. Furthermore, some authors have reported that there is no difference in survival by age at diagnosis [8,9]. In the present study, compared with patients aged between 31 and 50 years, patients aged ≤30 years have a significantly poor prognosis. Our findings are consistent with other population-based studies showing difference in survival by age at diagnosis [6,7].

Histology was found to be prognostic in breast cancer. Lobular cancer had a better prognosis than ductal cancer for separate or combined stages [11]. Mclaughlin et al. reported that lobular cancer had a better prognosis than ductal cancer for combined stages [17]. In the present study, for the histology, there was no significant difference between the two age groups.

Current guidelines consider breast cancer patients age <35 years at high risk of relapse and/or mortality and recommend adjuvant chemotherapy irrespective of the stage of their tumor [18]. Moreover, Kroman et al. [9] have clearly demonstrated the unfavorable effect of young age in patients having no adjuvant therapy. In our study, almost 70% of the women aged ≤30 years received adjuvant chemotherapy. Furthermore, one retrospective analysis on treatment outcome leads to the hypothesis that the endocrine effects of chemotherapy alone were insufficient for patients in the younger age group with endocrine-responsive tumors, for whom suppression of estradiol production might be essential [19]. On the other hand, endocrine therapies are not easy to offer to very young patients [20,21], and further investigations in this specific field are urgently needed [22].

## Conclusions

Compared with patients aged between 31 and 50 years, patients aged ≤30 years have a greater chance of having an endocrine-unresponsive tumor and a significantly poor prognosis. Pathological tumor size, lymph node metastasis, and histology have a similar distribution among the younger and the older cohorts, thus not supporting previous data indicating more advanced disease in younger patients at diagnosis of operable disease.
